# Association between pulmonary ventilatory function and mild cognitive impairment: A population-based study in rural China

**DOI:** 10.3389/fpubh.2022.1038576

**Published:** 2022-11-02

**Authors:** Cuiying Gu, Mingfeng Ma, Jiahui Xu, Wei Yuan, Ruixue Li, Hui Guo, Hanshu Gao, Wenjing Feng, Haiqiang Guo, Liqiang Zheng, Yao Zhang

**Affiliations:** ^1^Department of Epidemiology, School of Public Health, China Medical University, Shenyang, China; ^2^Ministry of Education-Shanghai Key Laboratory of Children's Environmental Health, School of Public Health, Shanghai Jiao Tong University School of Medicine, Shanghai, China; ^3^Department of Cardiology, Fenyang Hospital of Shanxi Province, Fenyang, China; ^4^Department of Health Statistics, China Medical University, Shenyang, China; ^5^Department of Ultrasound, Shengjing Hospital of China Medical University, Shenyang, China

**Keywords:** pulmonary function, mild cognitive impairment, cognition, cross-sectional study, rural

## Abstract

**Background:**

Mild cognitive impairment (MCI), a reversible intermediate state, plays an important role in the development and prevention of dementia. The relationship between pulmonary function and MCI risk has not yet been well-elucidated.

**Methods:**

We included 2,947 rural Chinese residents aged ≥35 years who were free from a history of stroke, dementia, or other brain diseases and measured pulmonary ventilatory function using calibrated spirometry according to the recommended method. MCI was assessed with the Montreal Cognitive Assessment-Basic for Chinese scale. Logistic regression models and restricted cubic splines with covariate adjustment were performed to explore the association between pulmonary function and MCI risk.

**Results:**

The prevalence of MCI increased with decreasing pulmonary function, from the lowest quartile to the highest quartile of pulmonary function: 63.9, 50.5, 43.8, and 43.6%, respectively. After adjustment for confounding factors, participants in the first quartile had a significantly increased risk of MCI (ORs, 1.691, 95% CI, 1.267–2.258), with the highest quartile as the reference. In the subgroup analysis, a significant association of pulmonary function and MCI was found in females and those with low physical activity. Meanwhile, we observed an L-shaped relationship between pulmonary function and MCI (*P*_non−linear_ = 0.032).

**Conclusions:**

Poor pulmonary function was associated with an increased risk of MCI among rural Chinese adults, and presented a non-linear relationship. These findings remind us of the need for early cognitive assessment in local populations with lower pulmonary function.

## Introduction

Dementia is one of the major public health problems and the leading cause of disability in elderly individuals worldwide (1). People with dementia in China account for ~25% of the global dementia population, imposing immense challenges on patients, families and public health care systems (2). Mild cognitive impairment (MCI), a prodromal stage of dementia, is defined as subjective cognitive decline in combination with objective memory impairment beyond normal age-related changes without apparent impact on daily living (3). In China, large percentages of the population, particularly those living in rural areas, suffer from cognitive dysfunction. Recent studies have reported that the prevalence of MCI was 26.5% among elderly adults in rural areas, compared with 12.2% in urban areas and an overall prevalence of 15.5% (4–6). Additionally, adults with MCI are generally considered to have a higher risk of progression to dementia than the normal population, with an annual conversion rate ranging from 10 to 20% and a rate of 60–100% over 5–10 years in the elderly (7, 8). Encouragingly, MCI is a potentially reversible state, and the rate of progression from MCI to dementia is similar to that of reversion from MCI to normal (9). Thus, in the absence of any curative treatment for dementia, early identification of individuals at risk for MCI is a key interventional target for dementia, and adopting effective preventive strategies may delay or reverse disease progression to postpone the onset of dementia, which has prompted researchers to pay more attention in recent decades.

In addition to well-recognized risk factors, impaired pulmonary function is becoming increasingly recognized as a predictor of cognitive performance (10). A recent meta-analysis of cohort studies demonstrated that people with poorer pulmonary function and those with respiratory disease may be a risk factor for dementia (11). Data retrieved from the AGES-Reykjavik Study revealed that lower pulmonary function measured in midlife predicted poor memory, processing speed, executive function, MCI and dementia 23 years later (12). Accumulating epidemiological studies have also illustrated that the global cognitive decline and a greater risk of dementia have a significant relationship to indicators with pulmonary function expressed as forced vital capacity (FVC), forced expiratory volume in 1 second (FEV1) and peak expiratory flow (PEF) (12–17). However, previous studies have either focused on the association of COPD with dementia or MCI, or have targeted community-dwelling participants with relatively higher education and socioeconomic backgrounds, whereas information about the relationship between pulmonary function and MCI in rural populations is scarce (14–17). Furthermore, most studies have used only single a measure of ventilatory capacity to evaluate the respiratory condition and have not considered the resistance encountered during breathing and the contribution of respiratory muscles. Our aim, taken together, was to examine the association between pulmonary function and MCI among rural adults in China and whether the association may be modified by vital covariates.

## Materials and methods

### Study population

All data were collected from a baseline survey in rural areas of Fuxin County, North China, from June to August 2019. Based on the demographic characteristics, participants were recruited from the eastern, southern, and northern townships to take part in our survey. The inclusion criteria were residents aged 35 and above who resided locally for at least 5 years and could sign a consent form during the investigation. Subjects were excluded if one of the following criteria were met: (1) were pregnant; (2) developed severe liver and renal failure; or (3) were unwilling to participate in this study. Finally, 4,689 participants were recruited. All participants gave their informed consent at the time of the examination and the Committee of China Medical University authorized the study protocol. The procedures followed were performed under the ethical standards of the responsible committee on human experimentation of China Medical University ([2018]083).

**Figure 1:**
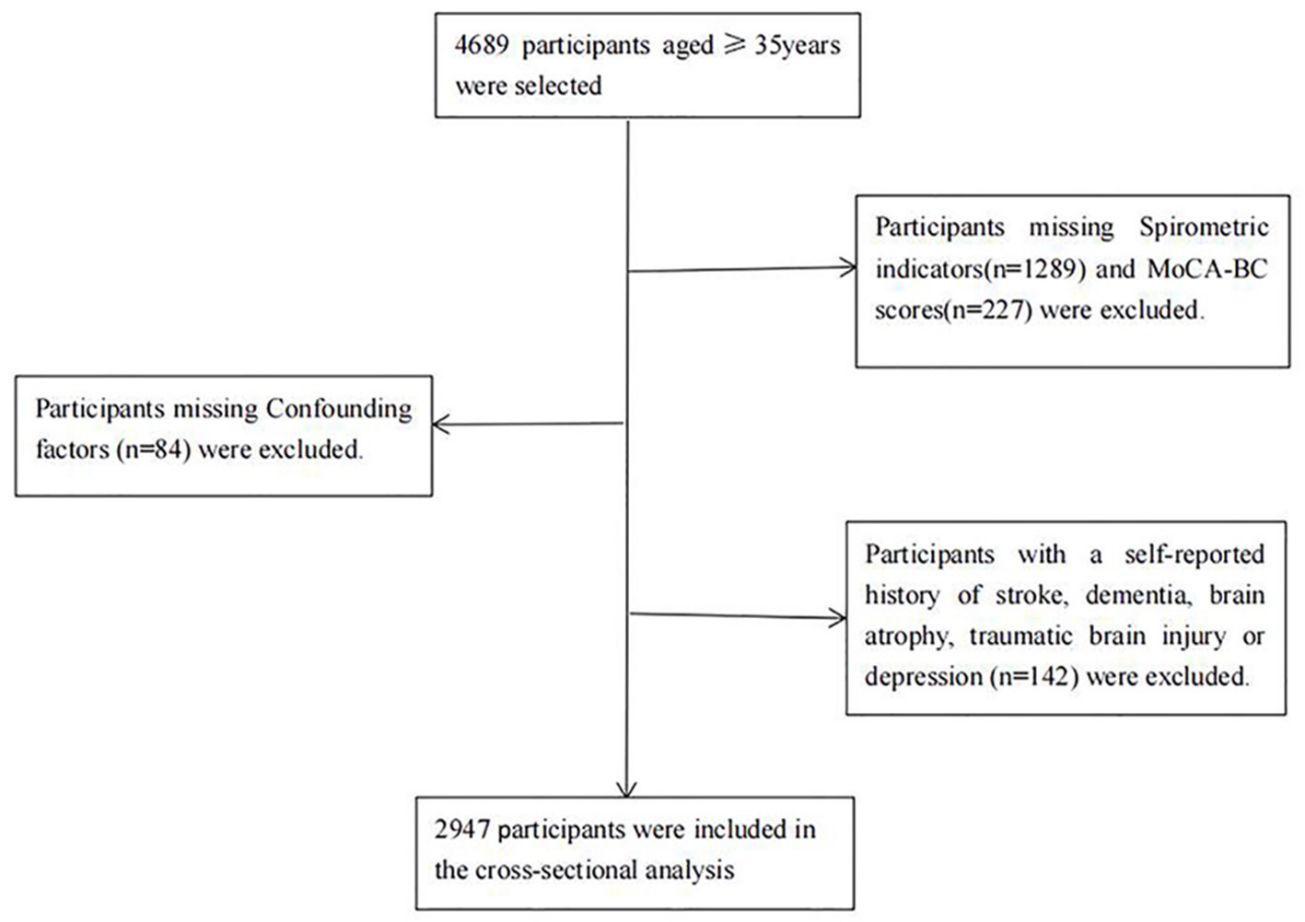
Flowchart of the participants included in the analysis. MoCA-BC, Chinese version of the Montreal Cognitive Assessment-Basic; MCI, mild cognitive impairment.

Of all 4,689 eligible participants, 1,600 dropped out due to missing information about spirometric indicators (*n* = 1,289), MoCA scores (*n* = 227) and confounding factors (*n* = 84). In addition, 142 participants with a self-reported history of stroke, depression, brain disease or dementia were excluded. In the end, Ultimately, 2,947 participants were enrolled in the analysis. A flowchart of the specific inclusion and exclusion criteria for participants in the current analysis is shown in Figure 1.

### Assessment of pulmonary function

Spirometry was performed by trained medical physicians with a calibrated Chest HI-101 (CHESTGRAPH Tokyo, Japan) according to the recommended method. Participants were asked to take a sitting position and put the disposable mouthpiece into the mouth, with lips sealed tightly around the mouthpiece, while pinching the nose, breathe calmly, then took the deepest breath possible to fill the lungs with air and blow as hard and as fast as possible (18). For each participant, at least three maneuvers were conducted to obtain repeatable results in which the corresponding best values were automatically recorded and confirmed by a technician. The best values of FVC, FEV1 and PEF were standardized into *z* scores by using the means and standard deviations computed from the entire study. According to previous studies (15, 16), we created a composite pulmonary function score by averaging the *z*-scores for FVC, FEV1, and PEF and then categorized them into four categories for the study.

### Assessment of MCI

The Montreal Cognitive Assessment (MoCA), published in 2005, has been proven more sensitive and reliable in MCI screening than other scales (19). For the present study, we used the Chinese version of the Montreal Cognitive Assessment-Basic (MoCA-BC) to detect MCI in rural Chinese adults with different education levels, which is a 30-point test covering nine cognitive domains: executive function, episodic memory, orientation, calculation, abstraction, delayed recall, visuospatial skill, naming and attention, with higher scores reflecting a higher level of global cognition (20). The optimal cutoff scores for MCI assessment by the MoCA-BC were 19 for individuals with 6 or fewer years of education, 22 for those with 7–12 years of education, and 24 for those with more than 12 years of education (21, 22). The MOCA scale was conducted by specially trained investigators.

### Assessment and definition of other variables

Relevant data on demographic variables (age, gender, ethnicity, education level), lifestyle factors (smoking, alcohol consumption), and history of disease were collected by face-to-face interview with a standardized questionnaire. Histories of disease were defined as self-reported and confirmed by medical records. Current smokers were defined as people who smoked at least one cigarette per day and continued for half a year. Alcohol ingestion was defined as at least three drinks every week for 6 months. Physical activity was rated three levels, low, moderate and high, based on the occupation engaged in.

Height and weight were measured using standardized procedures. BMI was calculated as weight (kg)/height (m)^2^. Hypertension was taken as an antihypertensive medication in the last 2 weeks, DBP ≥90 mmHg or SBP ≥140 mmHg (23). Diabetes mellitus was defined as fasting serum glucose concentration ≥7.0 mmol/L, using hypoglycemic drugs or insulin, or self-reported diabetes diagnosis by a physician or other health professionals (24).

### Statistical analysis

Continuous variables are represented as the mean ± standard deviation (SD) or medians (interquartile ranges), and categorical variables are reported as frequencies (percentages). Analysis of variance or the Kruskal–Wallis rank-sum test for continuous variables, respectively, while the chi-square test for categorical variables was used to compare differences in basic characteristics of different pulmonary function quartile groups. In addition, baseline characteristics comparisons between MCI and non-MCI were also made using Student's *t*-test for continuous variables or the chi-square test for categorical variables.

We examined associations of each pulmonary function parameter (FVC, FEV1, PEF) and MCI. Binary logistic regression models were used to estimate the odds ratios (ORs) and 95% confidence intervals (CIs) for composite pulmonary function and MCI. The composite pulmonary function score was assessed as quartiles 1–4 and quartile 1 vs quartiles 2–4. Three models were assessed, each building upon the prior model. Model 1 was unadjusted, model 2 was further adjusted for age and sex, and model 3 controlled for the variables in Model 2 plus body mass index (BMI), marital status, ethnicity, education level, smoke exposure, alcohol consumption, physical activity, hypertension, diabetes, histories of coronary heart disease (CHD), and chronic obstructive pulmonary disease (COPD). Tests for trends across quartiles were examined using ordinal values in separate models.

Moreover, we used restricted cubic splines (RCS) based on logistic regression models to visualize the relationship between pulmonary function and MCI on a continuous scale by adjusting the same confounders, with knots placed at the 10th, 50th, and 90th percentiles of the *z*-score of composite pulmonary function distribution. The threshold level for each section was determined using likelihood-ratio tests and bootstrap resampling methods. We further applied a piecewise regression model to examine the specific relationship between composite pulmonary function and MCI.

To assess relevant factors that had a modifying effect on the relationship between pulmonary function and MCI, we further conducted subgroup analyzes with study subjects stratified by sex, age (<65 or ≥65 years), smoking status (yes or no) and low physical activity (yes or no), respectively. We performed sensitivity analyzes to test the robustness of the results by repeating the analysis after excluding participants with respiratory symptoms (*n* = 176), histories of respiratory diseases, including COPD (*n* = 61), asthma (*n* = 4), pneumonia (*n* = 5), and other chronic lung diseases (*n* = 2). Subjects with occupational exposure (*n* = 176) or current smokers (*n* = 980) were also excluded.

The RCS analysis and threshold effect analyzes were conducted in R software, version 4.2.0 (http://www.R-project.org/) and other statistical analyzes were performed using IBM SPSS statistical software version 25.0 (SPSS Inc, USA). A 2-sided *P* value of <0.05 was considered statistically significant.

## Results

Out of 2,947 subjects with available data, 1,487 (50.5%) were observed to have MCI. The average age of the participants was 57.5 ± 9.8 years, and 1,939 (65.8%) were females. The characteristics of the study participants are described by different quartiles of pulmonary function in Table 1. Participants in the lowest quartile of pulmonary function tended to be slightly older, female, lower BMI, less educational attainment, lower physical activity, poorer MoCA scores and more likely to have hypertension, diabetes mellitus, and a more extensive history of CHD and COPD. At the same time, Supplementary Table S1 summarizes the differences in baseline characteristics among people with and without MCI. Compared to the participants without MCI, a higher proportion of those with MCI were generally older and significantly related to lower education levels, less physical activity, a higher prevalence of hypertension and diabetes mellitus, and self-reported CHD and COPD, and were more prone to be current smokers and drinkers. There were significant differences between the included and excluded groups in baseline characteristics except BMI (*P* = 0.359).

**Table 1:** Baseline characteristics of participants by quartiles of composite pulmonary function.

	**Quartile of PF ^a^**				***P-*value**
	**Q1 (*n* = 737)**	**Q2 (*n* = 736)**	**Q3 (*n* = 738)**	**Q4 (*n* = 736)**	
Age, mean (SD)	63.2 (9.1)	58.5 (8.8)	54.9 (9.3)	53.5 (8.8)	<0.001
BMI, mean (SD)	24.2 (3.7)	25.0 (3.8)	24.9 (3.5)	24.8 (3.6)	<0.001
Female, *n* (%)	621 (84.3)	620 (84.2)	526 (71.3)	172 (23.4)	<0.001
**Ethnicity**, ***n*** **(%)**
Han	472 (64.0)	439 (59.6)	486 (65.9)	485 (65.9)	0.055
Mongolian	240 (32.6)	255 (34.6)	227 (30.8)	223 (30.3)	
Others	25 (3.4)	42 (5.7)	25 (3.4)	28 (3.8)	
**Education level**, ***n*** **(%)**
Primary school or below	417 (56.6)	327 (44.4)	230 (31.2)	145 (19.7)	<0.001
Junior high school	236 (32.0)	302 (41.0)	393 (53.3)	431 (58.6)	
Senior high school or above	84 (11.4)	107 (14.5)	115 (15.6)	160 (21.7)	
**Marital status**, ***n*** **(%)**
Married	605 (82.1)	663 (90.1)	694 (94.0)	692 (94.0)	<0.001
Others	132 (17.9)	73 (9.9)	44 (6.0)	44 (6.0)	
**Physical activity**, ***n*** **(%)**
Lower	296 (40.2)	207 (28.1)	176 (23.8)	142 (19.3)	<0.001
Moderate	429 (58.2)	509 (69.2)	531 (72.0)	532 (72.3)	
Higher	12 (1.6)	20 (2.7)	31 (4.2)	62 (8.4)	
**Smoking**, ***n*** **(%)**
Non-smoker	521 (70.7)	555 (75.4)	539 (73.0)	317 (43.1)	<0.001
Current smoker	168 (22.8)	140 (19.0)	164 (22.2)	338 (45.9)	
Ex-smoker	48 (6.5)	41 (5.6)	35 (4.7)	81 (11.0)	
**Drinking**, ***n*** **(%)**
Non-drinker	598 (81.1)	600 (81.5)	538 (72.9)	350 (47.6)	<0.001
Current drinker	91 (12.3)	103 (14.0)	161 (21.8)	320 (43.5)	
Ex-drinker	48 (6.5)	33 (4.5)	39 (5.3)	66 (9.0)	
Hypertension, *n* (%)	309 (41.9)	287 (39.0)	237 (32.1)	267 (36.3)	0.001
Diabetes, *n* (%)	100 (13.6)	100 (13.6)	78 (10.6)	65 (8.8)	0.008
CHD, *n* (%)	100 (13.6)	66 (9.0)	57 (7.7)	32 (4.3)	<0.001
COPD, *n* (%)	40 (5.4)	8 (1.1)	3 (0.4)	10 (1.4)	<0.001
Occupational exposure, *n* (%)	76 (10.3)	67 (9.1)	56 (7.6)	97 (13.2)	0.003
MoCA-BC, median (IQR)	19.0 (14.0, 23.0)	21.0 (16.0, 24.0)	22.0 (18.0, 26.0)	23.0 (19.0, 26.0)	<0.001
MCI, *n* (%)	471 (63.9)	372 (50.5)	323 (43.8)	321 (43.6)	<0.001

FVC, forced vital capacity; FEV1, forced expiratory volume in 1 s; PEF, peak expiratory flow; PF, composite pulmonary function; BMI, body mass index; CHD, coronary heart disease; COPD, chronic obstructive pulmonary disease; MCI, mild cognitive impairment; MoCA-BC, the Chinese version of the Montreal Cognitive Assessment-Basic.

a Q1: PF < −0.6386; Q2: −0.6386 ≤ PF < −0.0687; Q3: −0.0687 ≤ PF < 0.5913; Q4: ≥0.5913.

**Figure 2:**
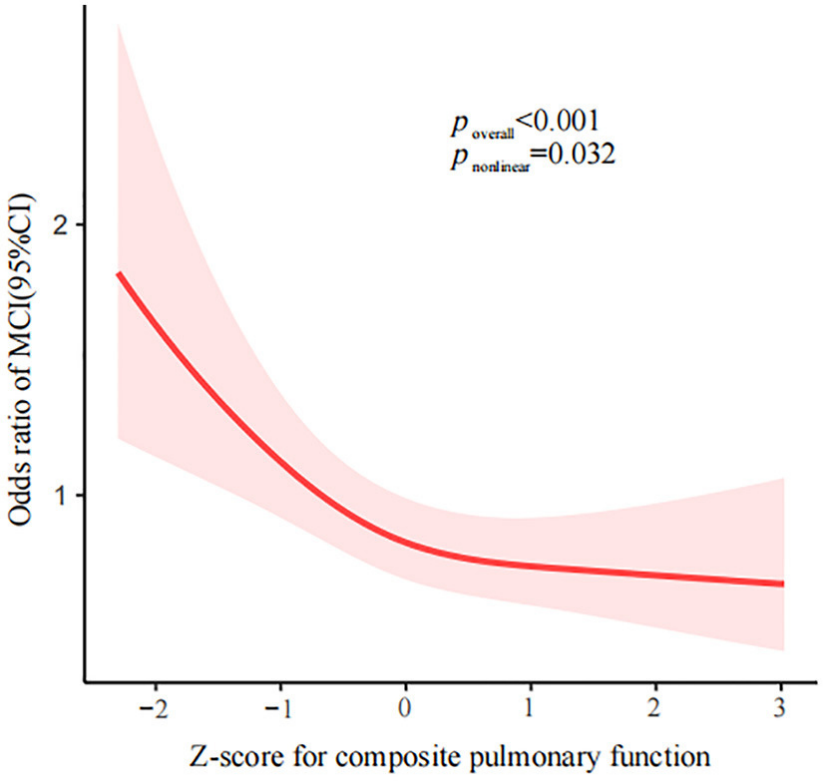
Association between *z*-score for composite pulmonary function and MCI using a restricted cubic spline regression. Multivariable adjusted odds ratios (ORs) and 95% CIs were derived from restricted cubic spline regression, with knots placed at the 10th, 50th, and 90th percentiles of *z*-score for composite pulmonary function. Solid red line represents multivariable adjusted odds ratios, with shaded part showing 95% confidence interval. Model adjusted for the same variables as model 3 in Table 2. MCI, mild cognitive impairment; ORs, odds ratio; CIs, confidence interval.

There were inverse associations between FVC, FEV1 and PEF and the risk of MCI (Supplementary Table S2). Consistently, the prevalence of MCI in the participants decreased with increasing pulmonary function, being 63.9% (471/737), 50.5% (372/736), 43.8% (323/738), and 43.6% (321/736) in the lowest to highest quartiles of pulmonary function, respectively (Table 1). The association between composite pulmonary function and the risk of MCI is presented in Table 2. Compared to the highest quartile of pulmonary function, the risk of MCI was 1.691 (95% CI, 1.267–2.258) in quartile 1, 1.280 (95% CI, 0.979–1.674) in quartile 2 and 1.127 (95% CI, 0.881–1.441) in quartile 3, with a significant trend test (*P* < 0.001) in the fully adjusted model. When quartiles 2–4 were further combined, the participants in the lowest quartile had a 42.4% higher risk of MCI than those in the upper quartiles (ORs, 1.424; 95% CI, 1.164–1.741). Additionally, we observed a non-linear relationship between pulmonary function and the risk of MCI (*P*_non − linear_ = 0.032, Figure 2). A cutoff point of *z*-score for composite pulmonary function = 0.214 yielded the best fitting model in a piecewise regression after multivariable adjustment. The risk of MCI declined rapidly with increasing pulmonary function until to the turning point and then started relatively flat. As shown in Table 3, an increase of 1 SD in pulmonary function was associated with an OR of 0.715 (95% CI, 0.604–0.845) for MCI, while there was no significant difference between pulmonary function and the risk of MCI when the *z* score of composite pulmonary function was ≥0.214 (ORs, 0.967; 95% CI, 0.821–1.131).

**Table 2:** Odds ratios (ORs) and 95% confidence intervals (CIs) for the relationship between composite pulmonary function and MCI^a^.

	**Model 1**		**Model 2**		**Model 3**	
	**OR (95% CI)**	***P-*value**	**OR (95% CI)**	***P-*value**	**OR (95% CI)**	***P-*value**
**Quartiles**
Q1 (<-0.6386)	2.289 (1.857–2.822)	<0.001	1.759 (1.328–2.330)	<0.001	1.691 (1.267–2.258)	<0.001
Q2 (−0.6386 to −0.0687)	1.321 (1.076–1.622)	0.008	1.294 (0.994–1.683)	0.055	1.280 (0.979–1.674)	0.071
Q3 (−0.0687 to −0.5913)	1.006 (0.819–1.236)	0.953	1.135 (0.892–1.446)	0.303	1.127 (0.881–1.441)	0.342
Q4 (≥0.5913)	1.000 (Ref.)		1.000 (Ref.)		1.000 (Ref.)	
*P* for trend ^b^		<0.001		<0.001		<0.001
**Categories**
Q1 (<-0.6386)	2.081 (1.752–2.472)	<0.001	1.471 (1.210–1.789)	<0.001	1.424 (1.164–1.741)	0.001
Q2–Q4 (≥-0.6386)	1.000 (Ref.)		1.000 (Ref.)		1.000 (Ref.)	

OR, odds ratio; CI, confidence interval; MCI, mild cognitive impairment; BMI, body mass index; CHD, coronary heart disease; COPD, chronic obstructive pulmonary disease; SD, standard deviation; Ref, reference.

Model 1: unadjusted; Model 2: Adjusted for age, sex; Model 3: Adjusted for age, sex, BMI, marital status, ethnicity, education level, smoke exposure, alcohol consumption, physical activity, hypertension, diabetes, histories of CHD and COPD.

a ORs and CIs were calculated using binary logistic regression models.

b Test for trend based on variable containing median value for each quartile.

**Table 3:** Threshold effect analyzes of association between composite pulmonary function and MCI using two piecewise regression models.

	**Adjust OR (95% CI)^a^**	***P*-value**
Logistic regression models	0.832 (0.745–0.926)	0.001
**Piecewise regression models**
PF < 0.214	0.715 (0.604–0.845)	<0.001
PF≥0.214	0.967 (0.821–1.131)	0.678
Likelihood ratio test		0.020

PF, composite pulmonary function; MCI, forced vital capacity.

a Adjusted for age, sex, BMI, marital status, ethnicity, education level, smoke exposure, alcohol consumption, physical activity, hypertension, diabetes, histories of CHD and COPD.

**Figure 3:**
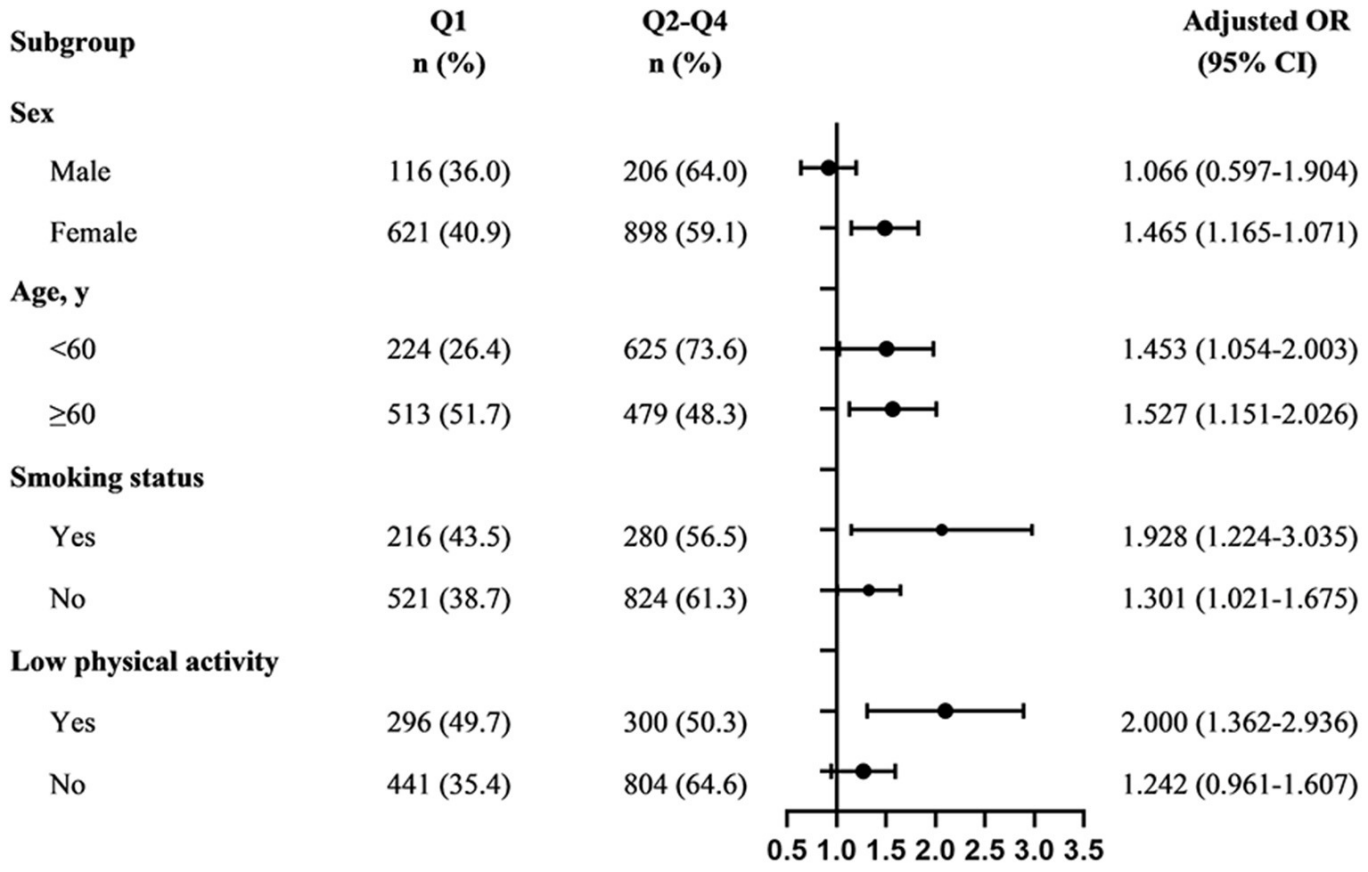
Subgroup analyzes of the association between composite pulmonary function and MCI among participants with *z*-score for composite pulmonary function <0.214. Q1: PF < −0.6386; Q2–Q4: ≥ −0.6386. Adjusted, if not stratified, for age, sex, BMI, marital status, ethnicity, education level, smoke exposure, alcohol consumption, physical activity, hypertension, diabetes, histories of CHD and COPD; MCI, mild cognitive impairment; ORs, odds ratio; CIs, confidence interval.

We further performed subgroup analyzes to explore potential modifier and interaction effects on the PF-MCI association in participants with *z*-score for composite pulmonary function <0.214. As shown in Figure 3, no significant evidence of the effects of the interactions between pulmonary function and important covariables on the risk of MCI was found (all *P* ≥ 0.05). Notably, we only found a relationship between lower pulmonary function and a higher risk of MCI in the female group and those with low physical activity, and the multivariate-adjusted ORs for MCI were 1.465 (95% CI, 1.165–1.071) and 2.000 (95% CI, 1.362–2.936), respectively. The risk of MCI for individuals with lower pulmonary function in non-smoker was higher (ORs, 1.301; 95% CI, 1.021–1.675), and even higher in smokers (ORs, 1.928; 95% CI, 1.224–3.035).

After exclusion of participants with respiratory symptoms, histories of respiratory diseases, occupational exposure and those who were current smokers, sensitivity analyzes yielded no any substantial differences from the main results (Supplementary Table S3).

## Discussion

In this population-based study of rural adults in Northeast China, we highlighted that poor pulmonary function was associated with a significantly increased risk of MCI and presented a non-linear relationship. Moreover, poor pulmonary function was associated with an elevated risk of MCI in females and those with low physical activity.

In normal human lungs, airflow depends on the expiratory driving pressure including the magnitude of expiratory muscle and elastic recoil as well as the size and viscoelastic properties of the lung and airway (25). FVC and FEV1/FVC have good sensitivity for diagnosing airflow limitation and are widely used relevant parameters for pulmonary function interpretation (26). PEF mainly reflects the caliber of the central airway and the pressure exerted by the expiratory muscles (27). These indicators of ventilator capacity are usually evaluated jointly in clinical diagnosis. Therefore, we used a composite pulmonary function indicator, including FVC, FEV1 and PEF, which took the thoracic volume, the resistance encountered during respiration and muscle strength into consideration, evaluating pulmonary function from different dimensions.

Evidence is converging as compromised lung health may be closely linked to cognitive performance. Burgraff et al. (28) found that a sustained decrease in cognitive function during 30 days of chronic hypercapnia in adult goats. Liang et al. (29) identified that children with hand, foot and mouth disease may have abnormalities in cognition, and respiratory function. A community-based cohort study involving 4,735 older Chinese adults showed that baseline COPD was independently associated with a 48.6% increased risk of MCI incidence (30). Another a prospective study including 1,425 cognitively normal participants aged 70–89 years, a diagnosis of COPD at baseline was associated with an increased risk of MCI, particularly na-MCI and presented a dose–response relationship between COPD duration of over 5 years at baseline and risk of MCI (31). Nevertheless, minimal data were available on the association between pulmonary function and MCI. In a previous population-based study, pulmonary function measured by FEV1/height^2^ exhibited that lower pulmonary function during midlife was associated with an increased likelihood of MCI in later life (12). Also, data from the Atherosclerosis Risk in Communities (ARIC), poor FEV1 and FVC were indicated to be associated with a higher risk of MCI (14). Recently, Wang et al. (15) reported that poor pulmonary function was linked to a nearly two-fold elevated risk of MCI. Our study, in line with the results mentioned above, held up the conclusion that lower pulmonary function was associated with a higher MCI risk. To note, we found that, adjusting for confounding factors, every increase of per SD in pulmonary function was associated with a 28.5% lower risk of MCI, whereas it was 22.0% in Reykjavik (OR, 0.78; 95% CI, 0.68, 0.89) (12). The ARIC study, which included 15,792 American adults, demonstrated that participants in the lowest quartile of FEV1% predicted (OR 1.34, 95% CI, 1.10–1.64) and FVC% predicted (OR 1.32, 95% CI 1.08–1.60) had significantly higher risks of MCI, after adjustment for potential confounders, with the highest quartile as the reference (14). Our finding, when pulmonary function was expressed in quartiles, that those in the lowest quartile were associated with an OR of 1.691 (95% CI, 1.267–2.258) for MCI compared with the highest quartile. Additionally, the prevalence of MCI for participants in the lowest quartile and in the highest quartile groups was 63.9 and 43.6%, respectively, which was much higher than the prevalence in Reykjavik (10.5% vs 4.8% for FEV1/height^2^) (12). Indeed, China has an unbalanced distribution of medical resources and a wide variation in medical standards between urban and rural areas (32). Residents in rural areas tend to experience disadvantages due to low income, poor living conditions, and reduced access to health services and low job satisfaction of primary health-care doctors (33, 34). In this regard, our findings may have important public health implications, as spirometry, which is easily measured, inexpensive, and highly reproducible in clinical practice, may be a potentially quick and easy-to-administer screening method for cognitive impairment to early identify people with poor pulmonary function who are at high risk of MCI and who might benefit from further neurodegenerative health assessments. However, further research to assess the feasibility and predictive ability of using pulmonary function tests as a screening tool for MCI is warranted.

In addition, we observed an L-shaped association between pulmonary function and MCI. There existed a threshold at which the risk of MCI decreased significantly with the increase in pulmonary function, and an additional increase in pulmonary function will have no substantial effects on the risk of MCI. These findings remind us of the need for early cognitive assessment in local populations with lower pulmonary function. We should be aware of potential cognitive deficits in individuals with poor pulmonary function that will likely further worsen their general health and quality of life. Village doctors, known as “gatekeepers” of the rural primary health-care system, play an indispensable role in ensuring and improving the health level of rural residents (34, 35). Therefore, they are advised to become acquainted with how to conduct brief cognitive assessment tests. In terms of underlying mechanisms, researchers have proposed that the lung and brain operate under common regulatory processes (36). The detrimental influence on brain is mediated *via* an intricate web of signaling involving hypoxia, oxidative stress, systemic inflammation, neurotransmitter function, or a combination of these processes (37–41). On the one hand, poor pulmonary function may reduce oxygen supply to the brain, affecting brain energy metabolism, thereby promoting the occurrence and development of ischemic brain injury, and exacerbating white matter lesions or lacunar infarction. On the other hand, brain ischemia induces oxidative stress and synaptic dysfunction that may result in oxidative stress–mediated damage, accelerating vascular damage and degenerative lesions. These pathophysiological processes may interact with each other, ultimately resulting in cognitive impairment.

A longitudinal study of older Chinese adults demonstrated that the positive association between pulmonary function and cognition was stronger in females (17). Similar to our results, participants with poor pulmonary function had higher odds of MCI in the female group and in the physically inactive group. The discrepancy in different sex was attributed to a reduction of estrogen after menopause (42). Moreover, females might have a higher risk of MCI than males because of differences in sensitivity to hypoxia or other physiological changes associated with pulmonary function decline (43). Another possible explanation was that the sample size in the male subgroup was much smaller than that in the female subgroup, which may have resulted in insufficient power to detect the significance of the difference. In this population-based study, we could not corroborate this speculation; thus, it is imperative to this hypothesis in the future. The evidence from population-based studies indicates that remaining an appropriate level of physical activity is not only associated with a lower risk of depression but may also may be an important protective factor of pulmonary function and cognitive health (44–46). Apart from the benefits of physical activity on respiratory muscle endurance and strength, it is increasingly accepted that regular physical activity has long-term systemic anti-inflammatory effects (44, 47). This may be closely related to the reduction of airway inflammation, the release and expression of neurotransmitters, brain-derived neurotrophic factor and other chemical factors in the brain, and the changes of cerebral hemodynamics (45, 48).

### Strengths and limitations

The major strength of our study is not only supported and extended existing evidence but also further found the non-linear relationship between pulmonary function and MCI, thereby further highlighting the need for early detection of cognitive performance in the lung-compromised population. Furthermore, integrated approach assessing pulmonary function, whereas previous studies have mainly focused on a single pulmonary function parameter.

However, inevitable limitations need to be borne in mind when interpreting the results. First, the associations of pulmonary function and MCI should be interpreted in the context of the cross-sectional design of the current study, in which the observed relationships are subject to potential confounding caused by unknown or unmeasured factors as well as the possibility of reverse causality, and thus cannot be interpreted as causal effects. Second, we only used a brief MoCA scale to define MCI, rather than a combination with comprehensive neuropsychological test assessments. Third, we must acknowledge that there might be selection bias due to a low response rate in spirometry. However, the pattern of age and sex distribution in subjects undergoing spirometry was similar to that in the entire subject group. Finally, all participants in this study were recruited from rural regions in Northeast China, which may limit the generalizability of our results.

In conclusion, poor pulmonary function was associated with an increased risk of MCI among rural Chinese adults, and presented a non-linear relationship. Further large prospective studies are needed to validate our findings. Overall, spirometry may be a simple and useful measure in primary health care practice, and individuals, especially those with poor lung function, should receive cognitive screening as early as possible to identify individuals at risk for early manifestations of dementia, which has important public implications for primary prevention targeted to rural adults.

## Data availability statement

The original contributions presented in the study are included in the article/Supplementary material, further inquiries can be directed to the corresponding author/s.

## Ethics statement

The studies involving human participants were reviewed and approved by the procedures followed were performed under the ethical standards of the responsible committee on human experimentation of China Medical University ([2018]083). The patients/participants provided their written informed consent to participate in this study.

## Author contributions

YZ and LZ foresaw this work and designed the overall research. JX, WY, RL, HuG, HG, and WF collected primary data and screened the data. CG performed the statistical analysis and drafted the manuscript. MM participated in data interpretation and critically revised the manuscript. HaG supervised the analysis. All authors contributed substantially to discussions of the content of this paper and agreed to the submitted version of the manuscript.

## Funding

This research was supported by funds from the National Nature Science Foundation of China [No. 82073645].

## Conflict of interest

The authors declare that the research was conducted in the absence of any commercial or financial relationships that could be construed as a potential conflict of interest.

## Publisher's note

All claims expressed in this article are solely those of the authors and do not necessarily represent those of their affiliated organizations, or those of the publisher, the editors and the reviewers. Any product that may be evaluated in this article, or claim that may be made by its manufacturer, is not guaranteed or endorsed by the publisher.

## Abbreviations

MCI: mild cognitive impairment
COPD: chronic obstructive pulmonary disease
FVC: forced vital capacity
FEV1: forced expiratory volume in 1 second
PEF: peak expiratory flow
PF: composite pulmonary function
MoCA: montreal cognitive assessment
MoCA-BC: montreal cognitive assessment-basic for Chinese
ORs: odds ratios
CIs: confidence interval
BMI: body mass index
CHD: coronary heart disease
RCS: restricted cubic splines.
