# Supplementary material for: Association between pulmonary ventilatory function and mild cognitive impairment: A population-based study in rural China
Source: Front Public Health. 2022 Nov 2;10:1038576. doi: 10.3389/fpubh.2022.1038576 (PMC9666756; doi:10.3389/fpubh.2022.1038576)
Supplement: Supplementary file 1 [file Data_Sheet_1.docx]

Supplementary Material

# Supplementary Tables

| **Supplementary Table 1. Baseline characteristics of participants** | | | | | | | |
| --- | --- | --- | --- | --- | --- | --- | --- |
|  |  | **Total** |  | **MCI** |  | **Non-MCI** | ***P-*value** |
|  |  | N=2947 |  | n=1487 |  | n=1460 |  |
| **Age, mean (SD)** | | 57.5 (9.8) |  | 60.6 (8.9) |  | 54.4 (9.7) | <0.001 |
| **BMI, mean (SD)** | | 24.7 (3.7) |  | 24.5 (3.7) |  | 24.9 (3.6) | 0.002 |
| **Sex, n (%)** | |  |  |  |  |  | <0.001 |
|  | **Female** | 1939 (65.8) |  | 919 (61.8) |  | 1020 (69.9) |  |
|  | **Male** | 1008 (34.2) |  | 568 (38.2) |  | 440 (30.1) |  |
| **Ethnicity, n (%)** | |  |  |  |  |  | 0.036 |
|  | Han | 1882 (63.9) |  | 952 (64.0) |  | 930 (63.7) |  |
|  | Mongolian | 945 (32.1) |  | 488 (32.8) |  | 457 (31.3) |  |
|  | Others | 120 (4.1) |  | 47 (3.2) |  | 73 (5.0) |  |
| **Education, n (%)** | |  |  |  |  |  | <0.001 |
|  | Primary school or below | 1119 (38.0) |  | 660 (44.4) |  | 459 (31.4) |  |
|  | Junior high school | 1362 (46.2) |  | 634 (42.6) |  | 728 (49.9) |  |
|  | Senior high school or above | 466 (15.8) |  | 193 (13.0) |  | 273 (18.7) |  |
| **Marital status, n (%)** | |  |  |  |  |  | 0.001 |
|  | Married | 2654 (90.1) |  | 1311 (88.2) |  | 1343 (92.0) |  |
|  | Others | 293 (9.9) |  | 176 (11.8) |  | 117 (8.0) |  |
| **Physical activity, n (%)** | |  |  |  |  |  | <0.001 |
|  | Lower | 821 (27.9) |  | 433 (29.1) |  | 388 (26.6) |  |
|  | Moderate | 2001 (67.9) |  | 1020 (68.6) |  | 981 (67.2) |  |
|  | Higher | 125 (4.2) |  | 34 (2.3) |  | 91 (6.2) |  |
| **Smoking, n (%)** | |  |  |  |  |  | <0.001 |
|  | Non-smoker | 1932 (65.6) |  | 899 (60.5) |  | 1033 (70.8) |  |
|  | Current smoker | 810 (27.5) |  | 467 (31.4) |  | 343 (23.5) |  |
|  | Ex-smoker | 205 (7.0) |  | 121 (8.1) |  | 84 (5.8) |  |
| **Drinking, n (%)** | |  |  |  |  |  | <0.001 |
|  | Non-drinker | 2086 (70.8) |  | 1000 (67.2) |  | 1086 (74.4) |  |
|  | Current drinker | 675 (22.9) |  | 376 (25.3) |  | 299 (20.5) |  |
|  | Ex-drinker | 186 (6.3) |  | 111 (7.5) |  | 75 (5.1) |  |
| **Hypertension, n (%)** | | 1100 (37.3) |  | 622 (41.8) |  | 478 (32.7) | <0.001 |
| **Diabetes, n (%)** | | 343 (11.6) |  | 199 (13.4) |  | 144 (9.9) | 0.003 |
| **CHD, n (%)** | | 255 (8.7) |  | 147 (9.9) |  | 108 (7.4) | 0.016 |
| **COPD, n (%)** | | 61 (2.1) |  | 43 (2.9) |  | 18 (1.2) | 0.002 |
| **Occupational Exposure, n (%)** | | 296 (10.0) |  | 137 (9.2) |  | 159 (10.9) | 0.130 |
| Abbreviations: BMI, body mass index; CHD, coronary heart disease; COPD, chronic obstructive pulmonary disease; MCI, mild cognitive impairment;  variables are presented as mean ± SD or n (%); *P* Value was calculated using Student’s t test for continuous variables and χ^2^ test was for categorical variables. | | | | | | | |

| **Supplementary Table 2. Association between different pulmonary function parameters and MCI ^a^** | | | | | | |
| --- | --- | --- | --- | --- | --- | --- |
|  |  | **Quartiles of pulmonary function, OR (95% CI)** | | | | ***P* for trend ^b^** |
|  |  | **Q1** | **Q2** | **Q3** | **Q4** |  |
| **Z-score for** **FVC** | |  |  |  |  |  |
|  | Model1 | 2.045 (1.663 - 2.514) | 1.148 (0.935 - 1.410) | 0.923 (0.752 - 1.132) | 1.000(Ref.) | <0.001 |
|  | Model2 | 1.549 (1.182 - 2.030) | 1.077 (0.835 - 1.389) | 0.971 (0.766 - 1.230) | 1.000(Ref.) | <0.001 |
|  | Model3 | 1.515 (1.148 - 2.000) | 1.067 (0.823 - 1.383) | 0.951 (0.747 - 1.210) | 1.000(Ref.) | 0.002 |
| **Z-score for FEV1** | |  |  |  |  |  |
|  | Model1 | 2.230 (1.809 - 2.749) | 1.367 (1.112 - 1.681) | 0.924 (0.753 - 1.134) | 1.000(Ref.) | <0.001 |
|  | Model2 | 1.535 (1.162 - 2.028) | 1.263 (0.974 - 1.636) | 0.981 (0.774 -1.245) | 1.000(Ref.) | <0.001 |
|  | Model3 | 1.488 (1.119 - 1.979) | 1.251 (0.961 - 1.627) | 0.980 (0.770 - 1.249) | 1.000(Ref.) | 0.001 |
| **Z-score for PEF** | |  |  |  |  |  |
|  | Model1 | 2.281(1.851 - 2.810) | 1.407 (1.146 - 1.727) | 1.174 (0.956 - 1.443) | 1.000(Ref.) | <0.001 |
|  | Model2 | 1.667 (1.302 - 2.135) | 1.306 (1.036 - 1.647) | 1.214 (0.970 - 1.519) | 1.000(Ref.) | <0.001 |
|  | Model3 | 1.583 (1.229 - 2.040) | 1.298 (1.025 - 1.643) | 1.201 (0.956 - 1.508) | 1.000(Ref.) | 0.001 |
| Abbreviations: OR: odds ratio; CI: confidence interval; MCI, mild cognitive impairment; BMI, body mass index; CHD, coronary heart disease; COPD, chronic obstructive pulmonary disease; SD, standard deviation; Ref, reference.  Model1: unadjusted; Model2: Adjusted for age, sex; Model3: Adjusted for age, sex, BMI, marital status, ethnicity, education level, smoke exposure, alcohol consumption, physical activity, hypertension, diabetes, histories of CHD and COPD;  ^a^ ORs and CIs were calculated using binary logistic regression models.  ^b^ Test for trend based on variable containing median value for each quartile. | | | | | | |

| **Supplementary Table 3.** Odds ratios (ORs) and 95% confidence intervals (CIs) for the relationship between PF and MCI excluding participants participants with respiratory symptoms, a history of respiratory disease, smoking, and occupational exposure (n=1543). ^a^ | | | | | | | | | |
| --- | --- | --- | --- | --- | --- | --- | --- | --- | --- |
|  |  | **Model** | |  | **Model2** | |  | **Model3** | |
|  |  | **OR (95% CI)** | ***P-*value** |  | **OR (95% CI)** | ***P-*value** |  | **OR (95% CI)** | ***P-*value** |
| **Quartiles** | |  |  |  |  |  |  |  |  |
|  | Q1 (<-0.6386) | 2.505 (1.808-3.472) | <0.001 |  | 1.742 (1.158-2.621) | 0.008 |  | 1.662 (1.097-2.518) | 0.017 |
|  | Q2 (-0.6386 to -0.0687) | 1.497 (1.091-2.054) | 0.013 |  | 1.278 (0.869-1.878) | 0.212 |  | 1.251 (0.847-1.848) | 0.260 |
|  | Q3 (-0.0687 to -0.5913) | 1.158 (0.842-1.594) | 0.366 |  | 1.251 (0.869-1.803) | 0.229 |  | 1.201 (0.830-1.737) | 0.331 |
|  | Q4 (≥0.5913) | 1.000(Ref.) |  |  | 1.000(Ref.) |  |  | 1.000(Ref.) |  |
|  | *P* for trend ^b^ |  | <0.001 |  |  | 0.008 |  |  | 0.017 |
| **Categories** | |  |  |  |  |  |  |  |  |
|  | Q1 (<-0.6386) | 2.016 (1.597-2.545) | <0.001 |  | 1.407 (1.087 - 1.822) | 0.009 |  | 1.377 (1.059-1.791) | 0.017 |
|  | Q2-Q4(≥-0.6386) | 1.000(Ref.) |  |  | 1.000(Ref.) |  |  | 1.000(Ref.) |  |
| Abbreviations: OR: odds ratio; CI: confidence interval; MCI, mild cognitive impairment; BMI, body mass index; CHD, coronary heart disease; COPD, chronic obstructive pulmonary disease; SD, standard deviation; Ref, reference.  Model1: unadjusted; Model2: Adjusted for age, sex; Model3: Adjusted for age, sex, BMI, marital status, ethnicity, education level, alcohol consumption, physical activity, hypertension, diabetes, history of CHD;  ^a^ ORs and CIs were calculated using binary logistic regression models.  ^b^ Test for trend based on variable containing median value for each quartile. | | | | | | | | | |
